# Polyglycine Strands Within a Collagen Triple Helix

**DOI:** 10.1002/bip.70127

**Published:** 2026-11

**Authors:** Wasin Tiarawut, Gabriel Saenz, Jinyi Yang, Ronald T. Raines

**Affiliations:** Department of Chemistry, Massachusetts Institute of Technology, Cambridge, Massachusetts, USA

**Keywords:** circular dichroism spectroscopy, collagen triple helix, collagen-mimetic peptides, polyglycine, polyproline II helix

## Abstract

The collagen triple helix is the most abundant protein structural motif in animals. It tolerates any amino acid in its Xaa and Yaa positions but demands glycine (Gly) at every third position, where the residue packs into the sterically forbidding core of the triple helix and donates the recurring interstrand N–H⋯O=C hydrogen bond that stitches the three strands together. (2*S*)-Proline (Pro) and (2*S*,4*R*)-4-hydroxyproline (Hyp) are enriched far above their proteome-wide abundance because they preorganize individual strands into the polyproline II conformation, lowering the entropic cost of folding. We asked whether the triple helix is intrinsically stable enough to form from strands that carry no such preorganization, that is, from polyglycine. To offset the conformational entropy of a Gly-rich tract, we embedded Gly_6_ within flanking triple-helical (Pro-Hyp-Gly)_*n*_ clamps in Ac-(Pro-Hyp-Gly)_6_-Gly_6_-(Pro-Hyp-Gly)_5_-Pro-Hyp-NH_2_ and compared it with an otherwise identical peptide in which Gly_6_ was replaced by two [2-(2-aminoethoxy)ethoxy]acetic acid units, a flexible, length-matched spacer that lacks most of the regularly spaced backbone amides of Gly_6_. Circular dichroism spectroscopy revealed the diagnostic positive band at 225 nm. The molar ellipticity of the spacer-containing control was 86% of that of the Gly_6_-containing peptide, matching the 35/41 = 85% ratio of residues capable of triple-helix formation and consistent with the Gly_6_ segment contributing to the triple-helical signal. Thermal denaturation yielded a *T*_m_ of 46.8°C for the Gly_6_-containing peptide, 6.3°C higher than that of the matched spacer control. This added stability indicates that the polyglycine tract is not merely accommodated within the helix but contributes stabilizing interstrand hydrogen bonds. Together, these data indicate that three polyglycine strands can form a collagen triple helix when flanked by triple-helical clamps. This result is consistent with the idea that the triple-helical architecture predates the recruitment of Pro and Hyp, informs the evolutionary origin of collagen, and offers a blunt-ended, symmetric scaffold for designing peptidic triple helices.

## Graphical Abstract

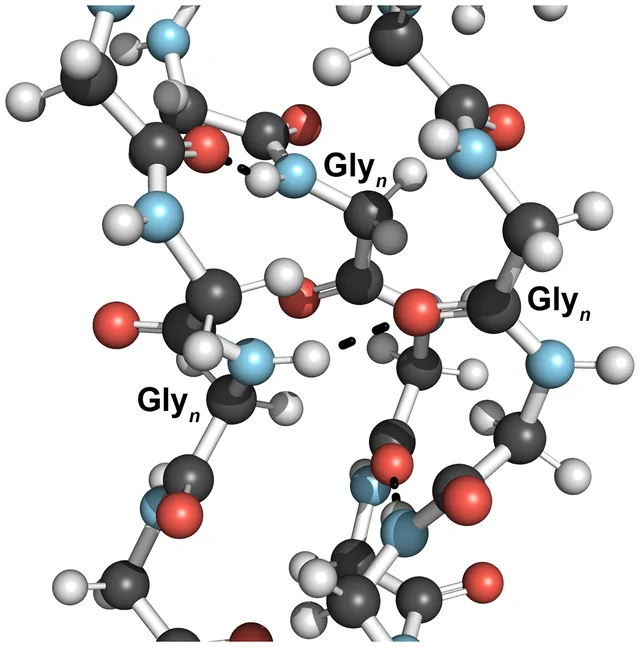

## 1 | Introduction

Collagen has been the dominant structural protein of animals for at least 200 million years and constitutes roughly one-third of the protein in the human body [1–3]. Its defining feature is an elegant motif in which three parallel, left-handed polyproline II (PPII) helices wind about a common axis, staggered by one residue, to form a right-handed triple helix [4–6]. The tight packing of the three strands against the superhelical axis imposes a single, absolute sequence requirement: every third residue of each strand must be glycine, yielding the repeating (Xaa-Yaa-Gly)_*n*_ motif.

Glycine is obligatory for two reasons. First, it is small. The α-carbon of the Gly residue sits in the crowded core of the triple helix, where any side chain—of either the l or the d configuration—cannot be accommodated (Figure 1) [6–8]. Gly substitutions, even to l-alanine, are among the most damaging mutations in collagenopathies such as osteogenesis imperfecta [9]. Second, the backbone amide of each Gly donates an interstrand N–H⋯O=C(Xaa) hydrogen bond from each triplet, worth ΔΔ*G*°≈−2.0 kcal/mol to stability, the most abundant amide–amide hydrogen bond in the animal kingdom [10]. Supplementary C^α^–H⋯O=C interactions further buttress the assembly [11].

Although any residue is permitted at Xaa and Yaa, collagen is far from random at those positions. Pro and Hyp together account for 22% of residues in human collagen, compared with a proteome-wide proline abundance of 7% [12, 13]. This enrichment is not required by the helix geometry; rather, it lowers the free energy of folding by preorganizing each strand [14]. Through stereoelectronic effects, including gauche effects and n→π* interactions, as well as steric effects, the pyrrolidine ring pucker of Pro and its derivatives fixes the *ϕ* and *ψ* torsion angles near their triple-helical values, so that the entropic cost of backbone ordering is largely prepaid in the unfolded state [15–18]. In short, the stability of natural collagen relies heavily on Pro and Hyp residues eliminating rotational degrees of freedom.

This logic raises a fundamental question: is the triple helix intrinsically stable, or merely the product of Pro/Hyp preorganization? Put differently, if we remove every preorganizing residue and retain only the obligatory glycine, does the architecture survive? Glycine is the most conformationally flexible residue [19, 20], yet polyglycine is not conformationally indifferent: crystallographic and solution studies show that polyglycine populates an extended, left-handed helical conformation (polyglycine II) closely related to a PPII helix [21, 22]. Moreover, the main-chain *ϕ* and *ψ* angles at the Xaa, Yaa, and Gly positions of a collagen triple helix all fall within the extended PPII region of conformational space (Table 1 [19, 20]) and are readily accessible to glycine. In addition, all of the peptide bonds in collagen are trans (*ω*~180°), and the *ω* angle is better preorganized as trans in glycine than in Pro or Hyp, whose tertiary amides populate the cis (*ω* ~ 0°) isomer far more readily [23]. We therefore hypothesized that three polyglycine strands can form a collagen-like triple helix.

The obstacle is entropic, not geometric. Replacing Pro and Hyp with Gly restores the rotational freedom that Pro and Hyp were recruited to suppress, raising the entropic cost of folding a free (Gly-Gly-Gly)_*n*_ strand into a triple helix [24]. We reasoned that this cost can be offset by holding the three Gly_6_ segments in register and proximity, so that the organization of the polyglycine core occurs within a preassembled trimer rather than through independent association of three flexible Gly-rich strands.

Tethering one end of three collagen-mimetic peptides (CMPs) to an organic template (e.g., Kemp’s triacid, TREN, Tris, or a macrocycle) is well precedented [25–30]. Tethering both ends has not been achieved with covalent bonds. Instead, we employ triple-helical (Pro-Hyp-Gly)_*n*_ segments as self-assembling “clamps” that flank a central polyglycine tract. Because each cross-section contains one Pro, one Hyp, and one Gly, the three strands are staggered yet still form an extensive all-glycine core (Figure 1).

Beyond the demonstration itself, a polyglycine triple helix raises questions that natural collagen cannot answer. Because every residue would be glycine, the structure could in principle adopt perfect screw symmetry, with identical *ϕ*, *ψ*, and *ω* angles at every position—unlike a natural triple helix, in which the Xaa, Yaa, and Gly positions differ (Table 1). Would triple-helical polyglycine therefore reveal whether the Pro/Hyp torsion angles are truly optimal for the helix, or merely the best that conformationally constrained residues can achieve? What helical pitch (the proline-rich 7 residues in 2 turns versus the proline-poor 10 residues in 3 turns [1]) would an unconstrained triple helix prefer? The answers bear directly on theories of the evolutionary origin of collagen, including the possibility that a glycine-rich, proline-poor primordial sequence formed triple helices, and on the design of peptidic triple helices as macromolecular scaffolds.

## 2 | Materials and Methods

### 2.1 | General Experimental Procedures

#### 2.1.1 | Reagents

Commercial chemicals were of reagent grade or better and were used without further purification. FmocAEEA and FmocGly(Dmb)GlyOH were from AAPPTec (Louisville, KY). FmocGlyOH and TFA were from Chem-Impex (Wood Dale, IL). Rink Amide ProTide Resin (low-loading) was from CEM (Matthews, NC). FmocHyp(tBu)OH was from AK Scientific (Union City, CA). DIC, Oxyma [31], and 4-methylpiperidine were from Oakwood Chemical (Tampa, FL). FmocProOH, acetic anhydride, and TIS were from Sigma–Aldrich (St. Louis, MO). All other reagents were from Thermo Fisher Scientific (Waltham, MA).

### 2.2 | Conditions

All procedures were performed at ambient temperature (~23°C) and pressure (1.0 atm) unless indicated otherwise.

### 2.3 | Peptide Synthesis—General

This section was adapted from Dones et al. (2019) [32]. Peptides were synthesized with a Liberty Blue Automated Microwave Peptide Synthesizer from CEM. All peptides were synthesized following CEM standard methods for both microwave and coupling cycles. Standard solutions of an activator, DIC (0.5 M in DMF), an activator base, Oxyma (1.0 M in DMF), deprotection solution, 4-methylpiperidine (20% v/v in DMF), Fmoc-protected amino acids (0.2 M in DMF), and a capping solution (10% v/v acetic anhydride in DMF) were prepared for each synthesis.

#### 2.3.1 | Microwave-Assisted Deprotection

The microwave was set to 155 W at 75°C for 15 s, followed by 30 W at 90°C for 50 s.

#### 2.3.2 | Microwave-Assisted Coupling

The microwave was set to 170 W at 75°C for 15 s, followed by 30 W at 90°C for 225 s.

### 2.4 | Standard Coupling Cycle

Rink Amide ProTide Resin (1 equiv) was added to the CEM reaction vessel and allowed to swell for 5 min in DMF. The Fmoc group was removed using the standard deprotection solution and the microwave-assisted deprotection methods described above. The resin was then washed (4×), and Fmoc-protected amino acid (5 equiv) was added, followed by DIC (20 equiv) and Oxyma (40 equiv). Standard microwave-assisted coupling was performed with additional Fmoc-protected amino acids, and the resin was washed (2×) and drained. When double-coupling was required, the cycle was repeated without the deprotection step. After the last coupling, the N-terminal amino acid was deprotected and capped by treating with acetic anhydride.

### 2.5 | Cleavage and Precipitation

After capping, the resin was moved from the reaction vessel to a cleavage filter, washed with DCM (3×), and air-dried. Crude peptides were then cleaved from the resin using a cleavage cocktail composed of 95:2.5:2.5 TFA/H_2_O/TIS for 2 h. Peptide mixtures were filtered and precipitated in ice-cold diethyl ether. The peptides were collected by centrifugation, the supernatants were decanted, and the solid peptide was dissolved in 5 mL of 70:30 H_2_O/ACN. The solutions were frozen and lyophilized using a FreeZone benchtop instrument from Labconco (Kansas City, MO). The peptide was then purified from the crude mixture.

### 2.6 | Peptide Synthesis—Specific

CMP **1** was synthesized as Ac-(Pro-Hyp-Gly)_6_-Gly-Gly(Dmb)-Gly-Gly_3_-(Pro-Hyp-Gly)_5_-Pro-Hyp-NH_2_ by using FmocGly(Dmb)Gly-OH to install a temporarily backbone-protected Gly-Gly unit within the Gly_6_ tract. The Dmb group disrupts interstrand hydrogen bonding and suppresses on-resin aggregation of glycine-rich sequences [33]. The Dmb group is removed during cleavage following capping, and the peptide is precipitated to afford a crude product.

CMPs **2**–**4** were synthesized by a non-interrupted continuous method. After deprotection of the Fmoc-protected Rink Amide ProTide Resin, Fmoc-protected amino acids were coupled using either a single or a double standard coupling cycle until completion. The low nucleophilicity of Pro and Hyp required a double standard coupling cycle. Following capping with acetic anhydride, the peptide was cleaved from the resin and precipitated to afford a crude peptide product.

### 2.7 | Peptide Purification and Characterization

The crude peptide products were purified by preparative reversed-phase HPLC using an XSelect Peptide CSH C18 OBD Prep Column 130 Å, 5 μm, 19 × 250 mm from Waters (Milford, MA) and a 1260 Infinity II instrument from Agilent Technologies (Santa Clara, CA). Crude products were dissolved in the minimum amount of ACN and eluted using a linear ACN gradient (5%–80% v/v) in H_2_O containing TFA (0.1% v/v). After reviewing the initial chromatogram, the method was updated if necessary. Fractions containing purified peptide were pooled, lyophilized, and analyzed with analytical reversed-phase HPLC and MALDI–TOF mass spectrometry.

### 2.8 | Analytical HPLC

Analytical HPLC was performed on an Agilent 1260 Infinity II instrument equipped with a Waters XSelect CSH C18 column. Solvent A was 95:5 H_2_O/ACN containing TFA (0.1% v/v), and solvent B was 95:5 ACN/H_2_O containing TFA (0.1% v/v). Peptide purity was assessed using a linear gradient of 5%–40% v/v B over 15 min at a flow rate of 2.0 mL/min, with UV detection at 210 nm.

### 2.9 | MALDI–TOF Mass Spectrometry

MALDI–TOF mass spectrometry analyses were carried out with a microflex LRF mass spectrometer from Bruker (Billerica, MA). Peptides were analyzed by using a saturated solution of α-cyano-4-hydroxycinnamic acid in 30:70 ACN/H_2_O containing TFA (0.1% v/v).

### 2.10 | Circular Dichroism Spectroscopy

Peptide secondary structure was analyzed via circular dichroism (CD) spectroscopy. Peptides were dried under vacuum for ≥ 48 h before weighing and dissolving to approximately 75 μM in 10 mM sodium phosphate buffer, pH 7.5. To ensure consistency across samples, exact peptide concentrations were determined from absorbance at 205 nm using a Cary 60 UV–vis spectrophotometer from Agilent Technologies (Santa Clara, CA), with a molar absorptivity *ε* = 2780 M^−1^ cm^−1^ per amide bond [34]. The resulting solutions were heated to 65°C and then cooled to 4°C at 0.2°C/min. After ≥ 48 h, CD spectra were recorded in 1-mm path-length cuvettes at 11°C–13°C with a 1 nm bandpass at the MIT Biophysical Instrumentation Facility using a JASCO Model J-1500 CD spectrometer (Easton, MD). The signal was averaged for 4 s during the wavelength scan. Peptide solutions were heated at 0.5°C/min while monitoring ellipticity at 225 nm. Molar ellipticity was calculated from the measured ellipticity using the equation: (*θ* × *m*)/(*c* × *l* × 10), where *θ* is the measured ellipticity (mdeg), *m* is the molecular weight of the peptide (g mol^−1^), *c* is the peptide concentration (mg mL^−1^), and *l* is the cuvette path length (cm). Mean residue ellipticity was calculated from the measured ellipticity using the equation: (*θ* × *m*)/(*c* × *l* × *nr* × 10), where nr is the number of amino acid residues in the peptide. Normalizing per residue rather than per mole allows CD signals from peptides of different lengths to be compared directly. All CD spectroscopy experiments were performed in triplicate. We determined the reported *T*_m_ values as the temperature at the d*θ*_225nm_/d*T* minimum, obtained from the smoothed first derivative of the thermal denaturation curve determined with a first-degree Savitzky–Golay smoothing algorithm [35]. Because a triple helix is a trimeric assembly, these *T*_m_ values are “apparent” and vary with peptide concentration; all peptides were compared at a similar concentration (near 75 μM).

## 3 | Results and Discussion

### 3.1 | Peptide Design and Synthesis

To test the hypothesis that three polyglycine strands can form a collagen-like triple helix, we designed four CMPs (Figure 2). In CMP **1**, a central tract of six glycine residues is flanked on each side by a triple-helical (Pro-Hyp-Gly)_*n*_ clamp. Four design choices merit comment. First, a Gly_6_ tract was chosen to correspond to an integral number of Xaa-Yaa-Gly repeats and provide a substantial (5 residues × 3 strands) all-glycine core (Figure 1). Second, the clamps are proline-rich CMP segments that fold robustly into a triple helix, thereby nucleating assembly, setting the one-residue stagger, and holding the three Gly_6_ tracts in register and proximity—the entropic subsidy described above. Third, both termini are capped as amides to eliminate the interstrand Coulombic repulsion that free, like-charged termini would impose across the three closely packed strands [36]. Fourth, the C terminus ends in Pro-Hyp rather than in Gly, because a C-terminal Gly is strongly destabilizing to the triple helix, as shown by Wennemers and co-workers in their analysis of frame-shift effects [37].

To isolate the contribution of the polyglycine backbone, we synthesized CMP **2**, in which the Gly_6_ tract is replaced by two [2-(2-aminoethoxy)ethoxy]acetic acid (AEEA) units. The AEEA_2_ spacer approximates the length of Gly_6_ but lacks most of the regularly spaced amide N–H donors and carbonyl acceptors of a polyglycine backbone and cannot form the same ladder of interstrand N–H⋯O=C hydrogen bonds. The AEEA units incur gauche effects that promote helicity and could invoke O⋯O repulsion, but these influences should be relatively minor. Thus, CMPs **1** and **2** share identical clamps and differ only in the chemical nature of the central spacer. Any difference in stability is likely to reflect interactions within the polyglycine tract.

Finally, we synthesized the two individual clamps as isolated peptides. CMPs **3** and **4** correspond, respectively, to the N-terminal and C-terminal clamps of CMPs **1** and **2**. As short, untethered CMPs, they can form triple helices only as separate homotrimers and serve two purposes: they establish the baseline behavior of a clamp in the absence of tethering, and, because they differ only in their C-terminal residue (Gly in CMP **3**, Hyp in CMP **4**), they provide an internal test of the destabilizing effect of a C-terminal glycine residue. HPLC traces and MALDI–TOF mass spectra are shown in Figures S1–S4.

### 3.2 | Circular Dichroism Supports Triple-Helix Formation

We annealed each peptide by cooling to 4°C and recorded its CD spectrum at 11°C–13°C (Figure 3A). All peptides displayed a maximum at 225 nm and a minimum near 198 nm, which are the canonical signatures of a PPII-based triple helix. By the magnitude of the 225-nm band, the triple-helical signal followed the order **1**>**2**>>**3**≈**4**: the two tethered, clamp-flanked peptides folded extensively, whereas the two short, untethered clamps folded only weakly.

The molar ellipticities of CMPs **1** and **2** are quantitatively concordant, with CMP **1** having a continuous triple helix that traverses the polyglycine tract. Because CMPs **1** and **2** carry identical clamps, they differ in triple-helix-competent residues only by the central spacer: CMP **1** presents 41 residues capable of triple-helix formation, whereas CMP **2** presents 35 (the two clamps; the AEEA units cannot participate), or 85% of CMP **1**. At 225 nm, the molar ellipticity of CMP **1** is *θ* = 90 ± 2 deg cm^2^ dmol^−1^, whereas that of CMP **2** is *θ* = 77 ± 2 deg cm^2^ dmol^−1^ (86% ± 3%). The near-identity of these two ratios means that the per-residue ellipticity is unchanged between the two peptides—that is, the six glycine residues of CMP **1** contribute to the 225-nm band just as the clamp residues do. This interpretation assumes that each glycine residue contributes to the 225-nm ellipticity to the same extent as a clamp residue; the close agreement between the measured ratio (86% ± 3%) and the residue count (85%) is consistent with that assumption. Were the Gly_6_ tract disordered, the ellipticity of CMP **1** would match that of CMP **2** (both reporting only the 35 clamp residues) rather than exceed it. The observed excess is therefore consistent with the Gly_6_ segment adopting the triple-helical conformation.

### 3.3 | Thermostability

We assessed thermostability by monitoring the 225-nm ellipticity as a function of temperature (Figure 3B). The transitions were cooperative, with midpoints in the order **1** > **2** > **4** > **3**. We report *T*_m_ values as the temperature of the minimum of the smoothed first derivative d*θ*_225nm_/d*T*, a model-free estimate that matches our operational definition of *T*_m_ and avoids assumptions about baseline symmetry. Reassuringly, this ranking and the CMP **1**–CMP **2** difference are preserved across all four fitting methods that we examined (Table S1), so our conclusions do not depend on this choice.

CMP **1** formed the most stable triple helix, with a *T*_m_ value of 46.8°C, which is 6.3°C higher than that of the matched control CMP **2** (40.5°C), though likely lower than that from replacing Gly_6_ with (ProHypGly)_2_ [1]. Because CMPs **1** and **2** are identical but for the central spacer, the 6.3°C increment shows that the polyglycine tract does not merely tolerate incorporation but actively adds stability relative to a flexible spacer of equal length—consistent with the formation of additional interstrand hydrogen bonds along the glycine backbone (Figure 1) rather than a mere end-to-end tether.

The two isolated clamps gave much weaker 225-nm CD signals and lower *T*_m_ values than CMP **1**, as expected for short, untethered CMPs. CMP **4** formed a more stable triple helix than CMP **3**, even though CMP **4** has one fewer residue (17 versus 18). The difference is attributable to the destabilizing C-terminal glycine of CMP **3**, consistent with the frameshift analysis by Wennemers and co-workers [37]. This internal control confirms that terminal composition, not merely chain length, governs stability in these constructs and reinforces our decision to terminate CMPs **1** and **2** with Pro-Hyp.

### 3.4 | Alternative Structures Are Unlikely

Could the added stability of the Gly_6_ segment arise from a competing secondary structure rather than from a triple helix? We think not. The most plausible alternative, a parallel β-sheet between two Gly_6_ strands, is disfavored on three grounds. First, glycine and proline have the lowest propensities to form a β-sheet [38–40], essentially mirroring the well-documented use of Gly/Pro-rich sequences by fibrous proteins to avoid β-sheet and amyloid formation [41]. Second, a competing two-stranded β-structure would leave the third Gly_6_ segment unsatisfied despite the clamps holding three strands in register at both ends. Third, the CD spectra show the positive 225-nm band characteristic of collagen triple helices rather than the spectral features expected for a β-sheet-rich assembly. The known preference of polyglycine to adopt an extended, PPII/polyglycine II-like conformation [21, 22] is consistent with its ability to be a strand within a triple helix. The simplest interpretation of the CD spectra, the concordant ellipticities, and the cooperative thermal transitions is therefore that three Gly_6_ units can form a collagen-like triple helix.

### 3.5 | Implications for Triple-Helix Geometry and Evolution

A genuine polyglycine triple helix would offer a unique window on triple-helix geometry. In a natural triple helix, residues in the Xaa, Yaa, and Gly positions adopt a similar *ω* angle but distinct *ϕ* and *ψ* angles (Table 1). In contrast, an all-glycine triple helix carries no such constraint and could in principle adopt a true screw symmetry. Are the “collagen” torsion angles in Table 1 the true optima for the architecture or merely the most readily achievable with conformationally locked residues?

This structural question connects to a deeper evolutionary one. Glycine is the most abundant α-amino acid detected in carbonaceous meteorites and has also been identified in comets [42–45], and glycine was likely among the most prevalent amino acids on prebiotic Earth [46–49] and perhaps the first incorporated into the genetic code [50–52]. Glycine residues are found at the Xaa and Yaa positions of human fibril-forming collagens, though not commonly (1.6% and 0.7%, respectively) [24]. That a triple helix can form from polyglycine supports a model in which the fold is intrinsically stable and could have arisen in a glycine-rich, proline-poor ancestor, with subsequent recruitment of l-proline and, ultimately, its posttranslational hydroxylation [53], raising thermostability toward body temperature [54, 55]. In this view, Pro and Hyp are optimizers of a preexisting architecture rather than its prerequisites.

## 4 | Conclusions

Three polyglycine strands, held in register by triple-helical (Pro-Hyp-Gly)_*n*_ clamps at both ends, form a structure with the CD signature and thermal cooperativity of a collagen triple helix, and the polyglycine tract contributes to—rather than detracts from—stability. Replacing the polyglycine with a flexible, amide-deficient spacer of equal length abolishes the effect, implicating the glycine backbone and its interstrand hydrogen bonds. Although an atomic-resolution structure is needed for proof, the results suggest that the triple-helical architecture is intrinsically stable without Pro or Hyp—consistent with an origin predating their evolution. In practice, the blunt, symmetric ends of a polyglycine triple helix enable tethering of both ends, thus yielding a new class of conformationally defined, minimalist triple-helical scaffolds.

## Supplementary Material

Supporting Information

Additional supporting information can be found online in the Supporting Information section. **Figure S1:** HPLC trace and mass spectrum of CMP **1**. Analytical HPLC trace (left) and MALDI–TOF mass spectrum (right) of purified peptide Ac-(Pro-Hyp-Gly)_6_-Gly_6_-(Pro-Hyp-Gly)_5_-Pro-Hyp-NH_2_ (CMP **1**). RT, 5.91 min. MALDI–TOF *m/z* [M + Na]^+^ expected, 3572.60; found, 3572.79. **Figure S2:** HPLC trace and mass spectrum of CMP **2**. Analytical HPLC trace (left) and MALDI–TOF mass spectrum (right) of purified peptide Ac-(Pro-Hyp-Gly)_6_-AEEA_2_-(Pro-Hyp-Gly)_5_-Pro-Hyp-NH_2_ (CMP **2**). RT, 6.43 min. MALDI–TOF *m/z* [M + Na]^+^ expected, 3520.62; found, 3520.59. **Figure S3:** HPLC trace and mass spectrum of CMP **3**. Analytical HPLC trace (left) and MALDI–TOF mass spectrum (right) of purified peptide Ac-(Pro-Hyp-Gly)_6_-NH_2_ (CMP **3**). RT, 5.13 min. MALDI–TOF *m/z* [M + Na]^+^ expected, 1684.76; found, 1684.66. **Figure S4:** HPLC trace and mass spectrum of CMP **4**. Analytical HPLC trace (left) and MALDI–TOF mass spectrum (right) of purified peptide Ac-(Pro-Hyp-Gly)_5_-Pro-Hyp-NH_2_ (CMP **4**). RT, 5.14 min. MALDI–TOF *m/z* [M + Na]^+^ expected, 1627.74; found, 1627.93. **Figure S5:** Smoothed thermal denaturation curves and first derivative curves for CMPs **1**–**4**. Smoothed thermal denaturation curves (left) and smoothed first derivative curves (right) for CMPs **1**–**4**. Unsmoothed curves are shown as dashed lines. Parameters used: window_size = 21, poly_order = 1. The concentrations from *A*_205 nm_ measurements were CMP **1**, 47 μM; CMP **2**, 58 μM; CMP **3**, 102 μM; CMP **4**, 92 μM. **Table S1:** *T*_m_ Values (°C) calculated from the data in Figure 3B by Different Methods. *T*_m_ Values (°C) Calculated from the Data in Figure 3B by Different Methods.

## Figures and Tables

**FIGURE 1 |:**
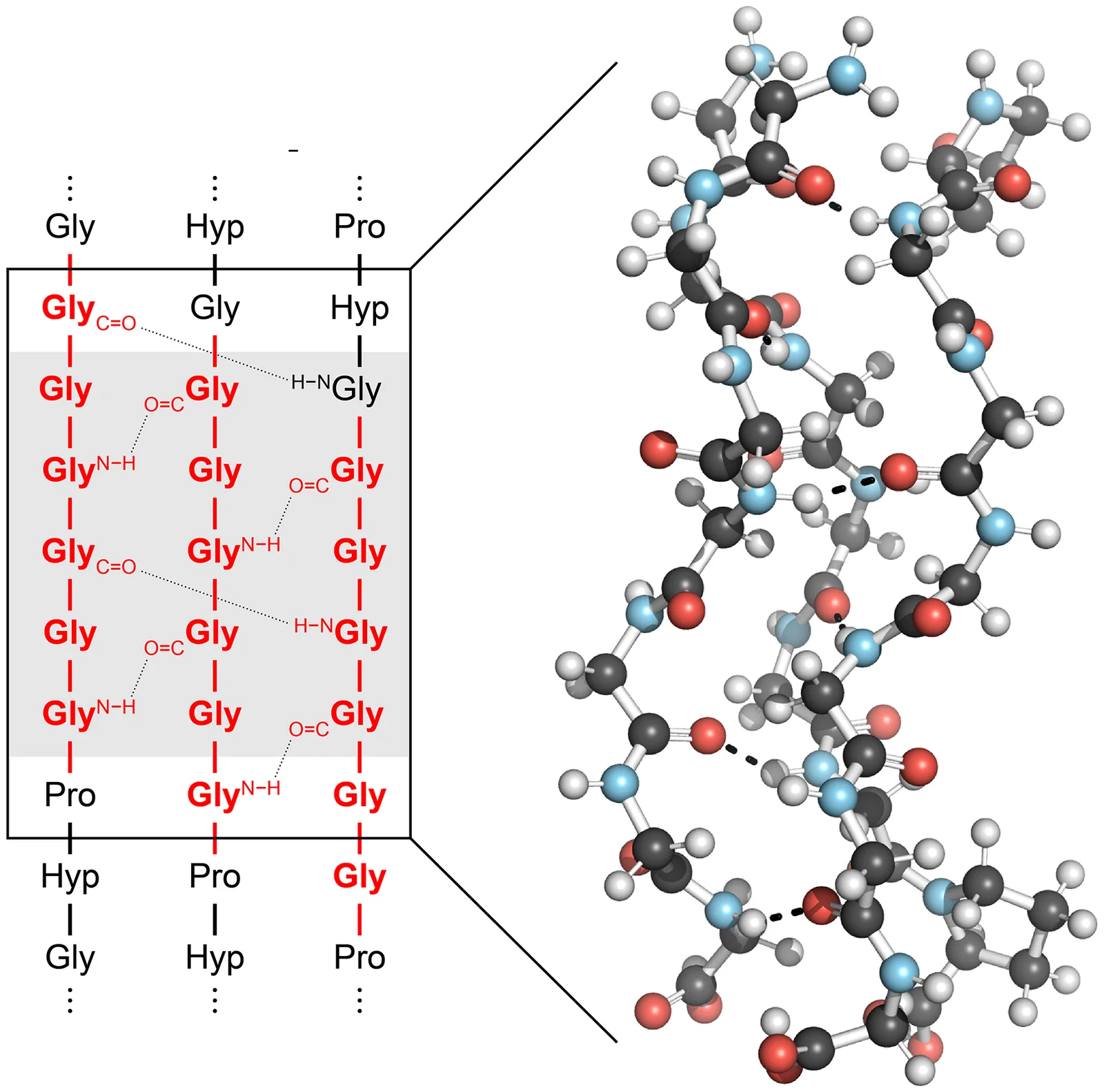
Illustrative molecular model of the core of triple-helical CMP **1**. Interstrand N–H⋯O=C hydrogen bonds are depicted as dashed lines. The shaded section comprises 15 glycine residues. The model was built by deleting side chains and terminal residues from a (Pro-Hyp-Gly)_11_ triple helix (PDB entry 1V7F [7]).

**FIGURE 2 |:**
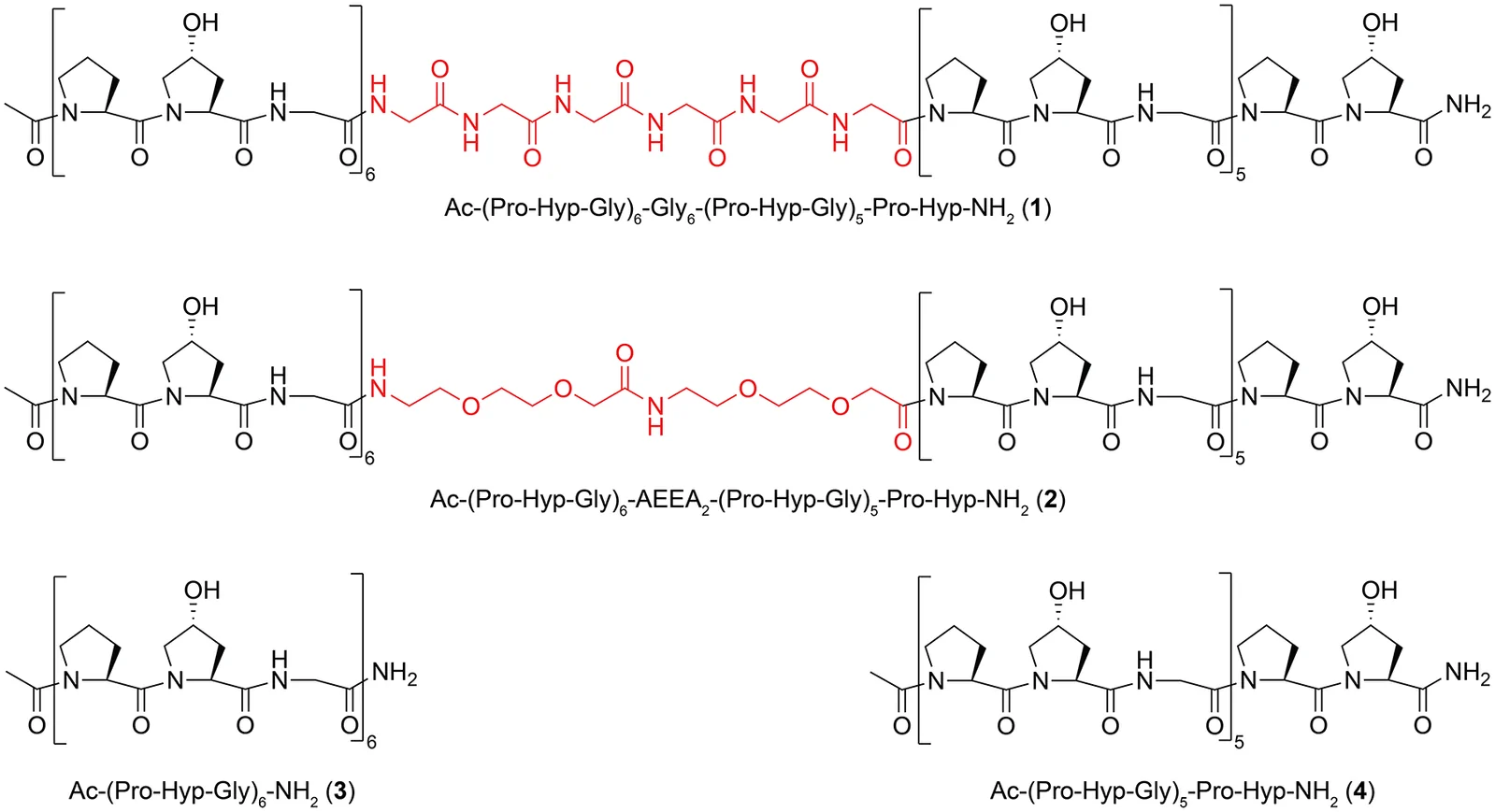
Structures of CMPs synthesized and analyzed in this work. CMP **1** contains a central Gly_6_ tract flanked by (Pro-Hyp-Gly)_*n*_ clamps. CMP **2** is the matched length-control in which Gly_6_ is replaced by AEEA_2_. CMPs **3** and **4** correspond to the isolated N-terminal and C-terminal clamps, respectively.

**FIGURE 3 |:**
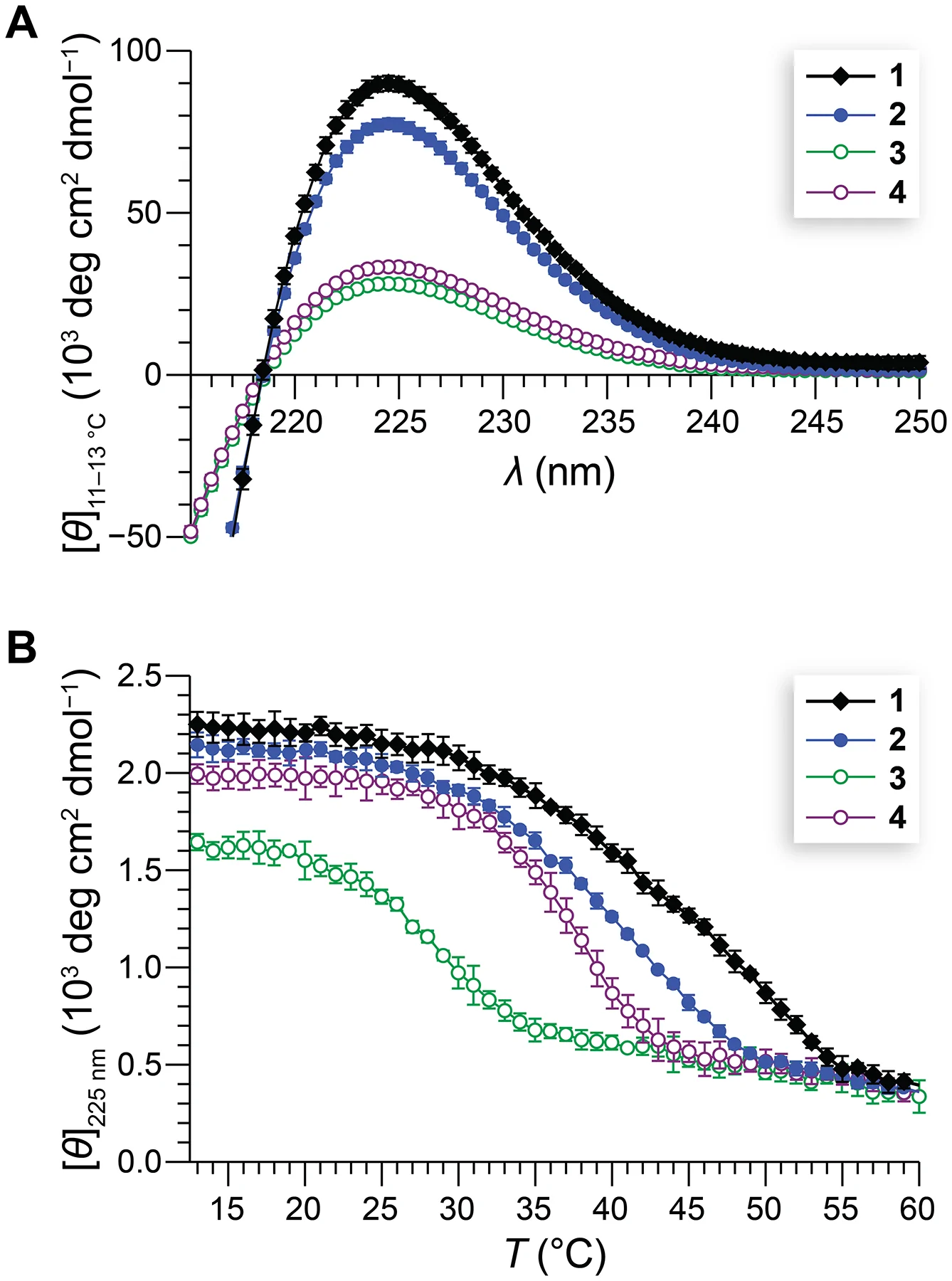
CD spectroscopy data for CMPs **1**–**4**. Experiments were performed in triplicate. (A) CD spectra of CMPs **1**–**4** (150 μM) at 11°C–13°C in 10 mM sodium phosphate buffer, pH 7.5. Values (mean ± SD) are molar ellipticities. (B) Thermal denaturation of triple-helical **1**–**4** (~75 μM) in 10 mM sodium phosphate buffer, pH 7.5, monitored at 225 nm. Values (mean ± SD) are mean residue ellipticities. The apparent *T*_m_ values are 46.8°C for CMP **1**, 40.5°C for CMP **2**, 28.8°C for CMP **3**, and 37.2°C for CMP **4** (Figure S5 and Table S1).

**TABLE 1 |:** Mean values of the main-chain torsion angles in a collagen triple helix [16].

Position	*ω*	*ϕ*	*ψ*
Xaa	176° ± 2°	−74° ± 3°	164° ± 4°
Yaa	175° ± 3°	−60° ± 4°	152° ± 3°
Gly	180° ± 2°	−72° ± 4°	176° ± 3°

*Note: ω*: C^α^_*i*_–C_*i*_–N_*i*+1_–C^α^_*i*+1_, *ϕ*: C_*i*−1_–N_*i*_–C^α^_*i*_–C_*i*_, *ψ*: N_*i*_–C^α^_*i*_–C_*i*_–N_*i*+1_.

## Data Availability

The data that support the findings of this study are available in the supporting information SI of this article.
