# Supplementary material for: Polyglycine Strands Within a Collagen Triple Helix
Source: Biopolymers. Author manuscript; Available in PMC 2026 Nov 1. (PMC13673506; doi:10.1002/bip.70127)
Supplement: Supplementary file 1 [file NIHMS2217642-supplement-Supporting_Information.pdf]

Polyglycine Strands within a Collagen Triple Helix

Wasin Tiarawut | Gabriel Saenz | Jinyi Yang | Ronald T. Raines

Correspondence: Ronald T. Raines (rtraines@mit.edu)

TABLE OF CONTENTS

Table of Contents S1
List of Abbreviations S2
Figure S1 | HPLC trace and mass spectrum of CMP **1** S3
Figure S2 | HPLC trace and mass spectrum of CMP **2** S3
Figure S3 | HPLC trace and mass spectrum of CMP **3** S4
Figure S4 | HPLC trace and mass spectrum of CMP **4** S4
Figure S5 | Smoothed thermal denaturation curves and first derivative curves for CMPs **1**–**4** S5
Table S1 | T_m Values (°C) Calculated from the Data in Figure 3B by Different Methods S6

List of Abbreviations

Ac	acetyl
ACN	acetonitrile
AEEA	[2-(2-aminoethoxy)ethoxy]acetic acid
CD	circular dichroism
CMP	collagen-mimetic peptide
DCM	dichloromethane
DIC	*N*,*N*′-diisopropylcarbodiimide
Dmb	2,4-dimethoxybenzyl
DMF	*N*,*N*-dimethylformamide
Fmoc	9-fluorenylmethoxycarbonyl
Gly	glycine
HPLC	high-performance liquid chromatography
Hyp	(2*S*,4*R*)-4-hydroxyproline
MALDI–TOF	matrix-assisted laser desorption/ionization–time-of-flight mass spectrometry
Oxyma	ethyl cyano(hydroxyimino)acetate
PBS	phosphate-buffered saline
PDB	Protein Data Bank
PPII	polyproline type II
Pro	(2*S*)-proline
RT	retention time
SD	standard deviation
*t*Bu	*tert*-butyl
TFA	trifluoroacetic acid
TIS	triisopropylsilane
T_m	apparent thermal-denaturation temperature
TREN	tris(2-aminoethyl)amine
Tris	tris(hydroxymethyl)aminomethane
UV–Vis	ultraviolet–visible spectroscopy

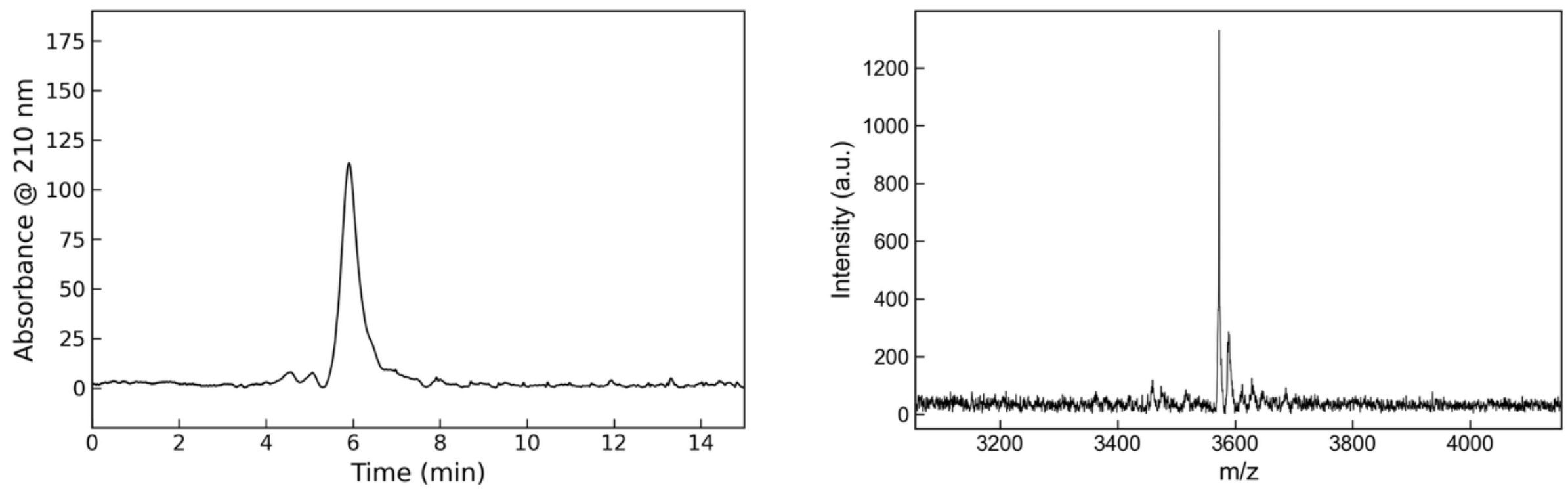

Figure S1 | Analytical HPLC trace (left) and MALDI–TOF mass spectrum (right) of purified peptide Ac-(Pro-Hyp-Gly)$_6$-Gly$_6$-(Pro-Hyp-Gly)$_5$-Pro-Hyp-NH$_2$ (CMP **1**). RT, 5.91 min. MALDI–TOF *m*/*z* [M + Na]$^+$ expected, 3572.60; found, 3572.79.

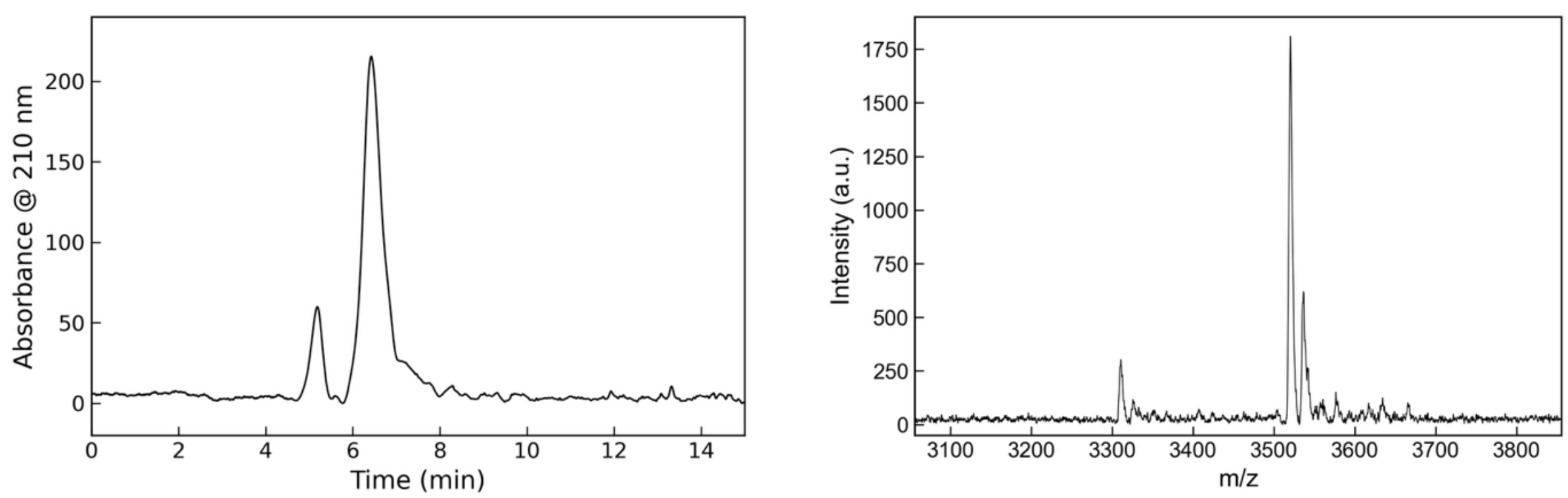

Figure S2 | Analytical HPLC trace (left) and MALDI–TOF mass spectrum (right) of purified peptide Ac-(Pro-Hyp-Gly)$_6$-AEEA$_2$-(Pro-Hyp-Gly)$_5$-Pro-Hyp-NH$_2$ (CMP **2**). RT, 6.43 min. MALDI–TOF *m*/*z* [M + Na]$^+$ expected, 3520.62; found, 3520.59.

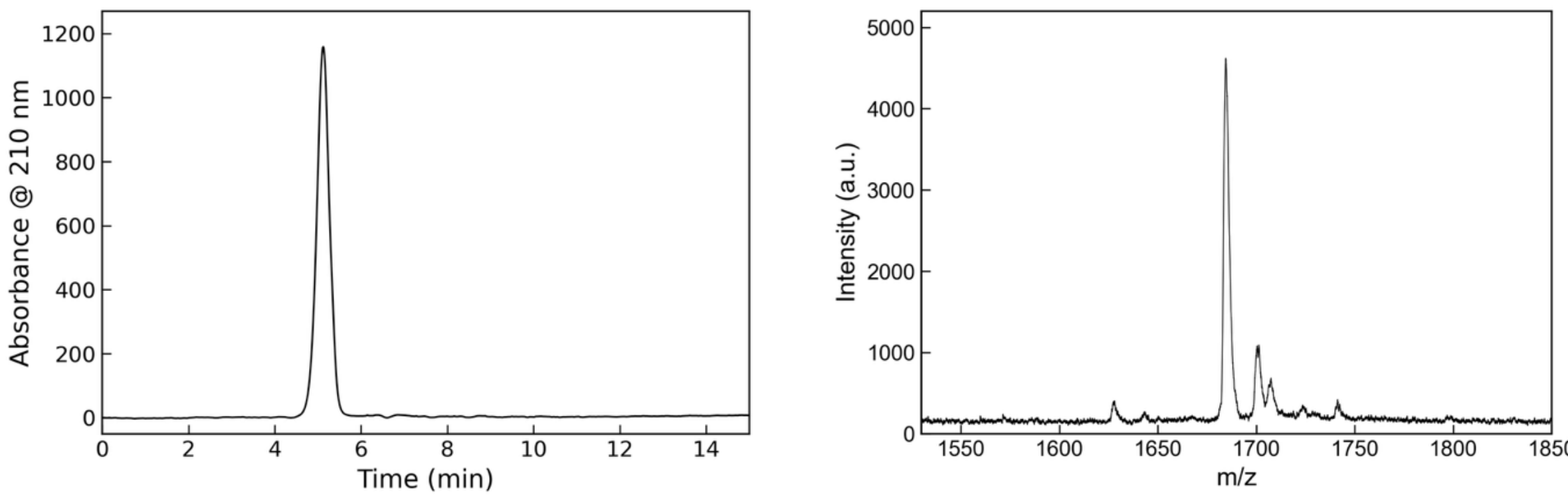

Figure S3 | Analytical HPLC trace (left) and MALDI–TOF mass spectrum (right) of purified peptide Ac-(Pro-Hyp-Gly)$_6$-NH_2 (CMP **3**). RT, 5.13 min. MALDI–TOF *m*/*z* $[M + Na]^+$ expected, 1684.76; found, 1684.66.

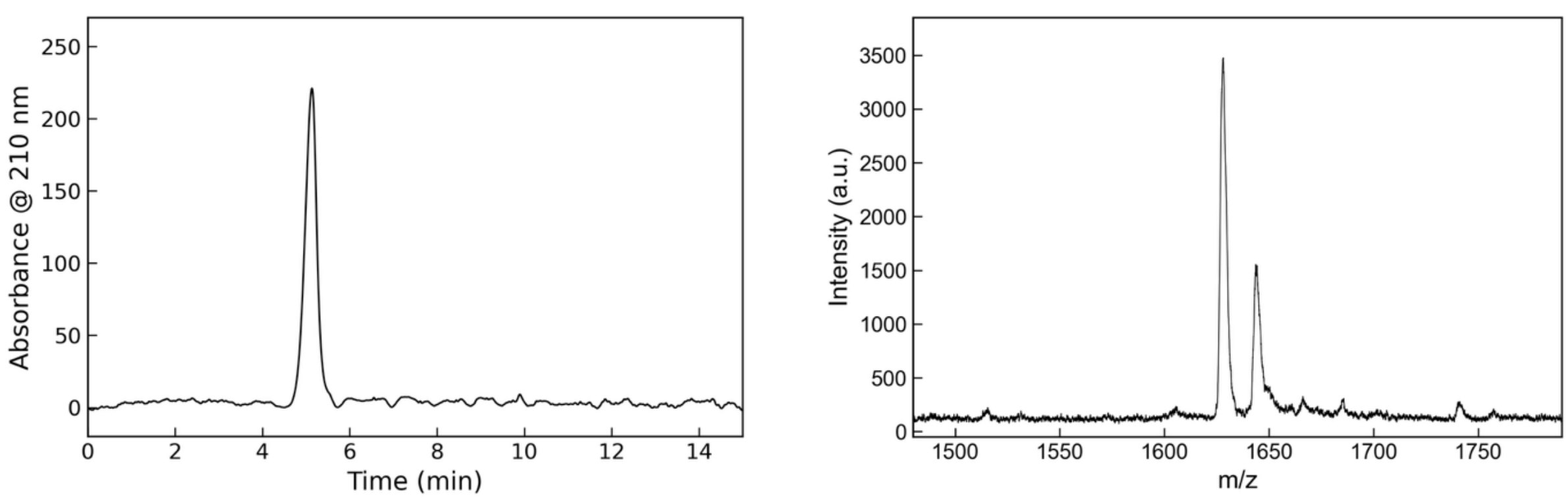

Figure S4 | Analytical HPLC trace (left) and MALDI–TOF mass spectrum (right) of purified peptide Ac-(Pro-Hyp-Gly)$_5$-Pro-Hyp-NH_2 (CMP **4**). RT, 5.14 min. MALDI–TOF *m*/*z* $[M + Na]^+$ expected, 1627.74; found, 1627.93.

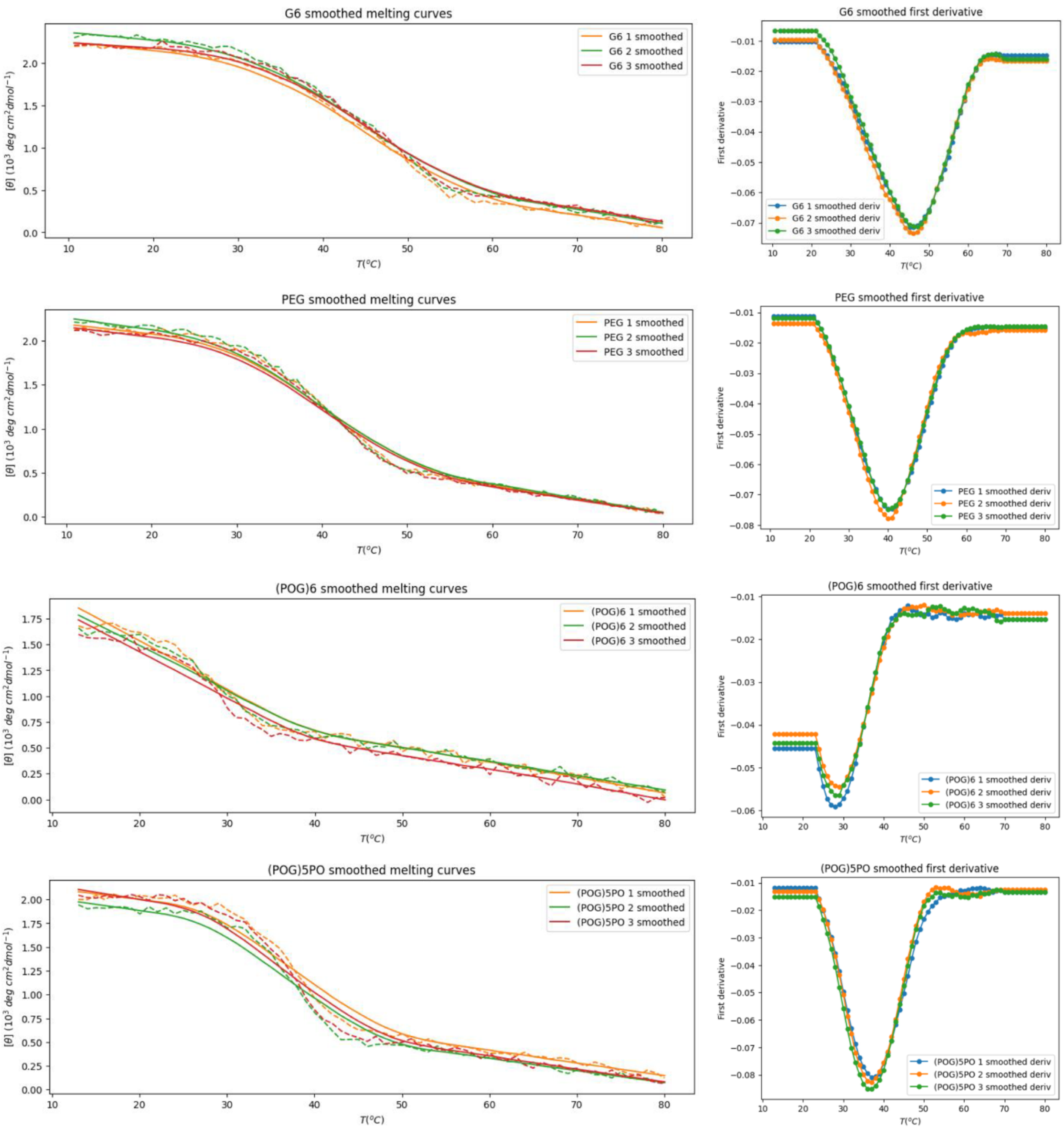

Figure S5 | Smoothed thermal denaturation curves (left) and smoothed first derivative curves (right) for CMPs **1**–**4**. Unsmoothed curves are shown as dashed lines. Parameters used: window_size = 21, poly_order = 1. The concentrations from $A_{205\ \mathrm{nm}}$ measurements were CMP **1**, 47 μM; CMP **2**, 58 μM; CMP **3**, 102 μM; CMP **4**, 92 μM.

Table S1 | T_m Values[a] (°C) Calculated from the Data in Figure 3B by Different Methods

Method	CMP			
	**1**	**2**	**3**	**4**
**Smoothed First-Derivative Minimum**	**46.8 ± 0.6**	**40.5 ± 0.0**	**28.8 ± 0.6**	**37.2 ± 0.6**
4-Parameter Sigmoid	45.3 ± 0.3	41.0 ± 0.3	21.5 ± 1.1	38.2 ± 0.4
5-Parameter Sigmoid Midpoint	45.4 ± 0.4	41.4 ± 0.2	30.9 ± 0.3	38.3 ± 0.4
5-Parameter Sigmoid Inflection Point	44.9 ± 0.4	38.6 ± 0.3	26.0 ± 0.3	35.8 ± 0.4

[a]Values are the mean ± SD for three replicates. The smoothed first-derivative minimum method selects the temperature among the measured values in the thermal denaturation experiment corresponding to the first-derivative minimum after smoothing, which can coincide across all three samples and result in an SD of 0.0.
