# Supplementary material for: Polyglycine Strands Within a Collagen Triple Helix
Source: Biopolymers. 2026 Oct 16;117(6):e70127. doi: 10.1002/bip.70127 (PMC13673506; doi:10.1002/bip.70127)
Supplement: Supplementary file 1 [file BIP-117-e70127-s001.docx]

**Polyglycine Strands within a Collagen Triple Helix**

**Wasin Tiarawut | Gabriel Saenz | Jinyi Yang | Ronald T. Raines**

**Correspondence:** Ronald T. Raines (rtraines@mit.edu)

**TABLE OF CONTENTS**

Table of Contents	S1

List of Abbreviations	S2

**Figure S1** | HPLC trace and mass spectrum of CMP **1**	S3

**Figure S2** | HPLC trace and mass spectrum of CMP **2**	S3

**Figure S3** | HPLC trace and mass spectrum of CMP **3**	S4

**Figure S4** | HPLC trace and mass spectrum of CMP **4**	S4

**Figure S5** | Smoothed thermal denaturation curves and first derivative curves for CMPs **1**–**4**	S5

**Table S1** | *T*_m_ Values (°C) Calculated from the Data in Figure 3B by Different Methods	S6

**List of Abbreviations**

Ac	acetyl

ACN	acetonitrile

AEEA	[2-(2-aminoethoxy)ethoxy]acetic acid

CD	circular dichroism 

CMP	collagen-mimetic peptide

DCM	dichloromethane

DIC	*N*,*N*′-diisopropylcarbodiimide

Dmb	2,4-dimethoxybenzyl

DMF	*N*,*N*-dimethylformamide

Fmoc	9-fluorenylmethoxycarbonyl

Gly	glycine

HPLC	high-performance liquid chromatography

Hyp	(2*S*,4*R*)-4-hydroxyproline

MALDI–TOF	matrix-assisted laser desorption/ionization–time-of-flight mass spectrometry

Oxyma	ethyl cyano(hydroxyimino)acetate

PBS	phosphate-buffered saline

PDB	Protein Data Bank

PPII	polyproline type II

Pro	(2*S*)-proline

RT	retention time

SD	standard deviation

*t*Bu	*tert*-butyl

TFA	trifluoroacetic acid

TIS	triisopropylsilane

*T*_m_	apparent thermal-denaturation temperature

TREN	tris(2-aminoethyl)amine

Tris	tris(hydroxymethyl)aminomethane

UV–Vis	ultraviolet–visible spectroscopy

|  |  |
| --- | --- |

**Figure S1** | Analytical HPLC trace (left) and MALDI–TOF mass spectrum (right) of purified peptide Ac-(Pro-Hyp-Gly)_6_-Gly_6_-(Pro-Hyp-Gly)_5_-Pro-Hyp-NH_2_ (CMP **1**). RT, 5.91 min. MALDI–TOF *m*/*z* [M + Na]^+^ expected, 3572.60; found, 3572.79.

|  |  |
| --- | --- |

**Figure S2** | Analytical HPLC trace (left) and MALDI–TOF mass spectrum (right) of purified peptide Ac-(Pro-Hyp-Gly)_6_-AEEA_2_-(Pro-Hyp-Gly)_5_-Pro-Hyp-NH_2_ (CMP **2**). RT, 6.43 min. MALDI–TOF *m*/*z* [M + Na]^+^ expected, 3520.62; found, 3520.59.

|  |  |
| --- | --- |

**Figure S3** | Analytical HPLC trace (left) and MALDI–TOF mass spectrum (right) of purified peptide Ac-(Pro-Hyp-Gly)_6_-NH_2_ (CMP **3**). RT, 5.13 min. MALDI–TOF *m*/*z* [M + Na]^+^ expected, 1684.76; found, 1684.66.

|  |  |
| --- | --- |

**Figure S4** | Analytical HPLC trace (left) and MALDI–TOF mass spectrum (right) of purified peptide Ac-(Pro-Hyp-Gly)_5_-Pro-Hyp-NH_2_ (CMP **4**). RT, 5.14 min. MALDI–TOF *m*/*z* [M + Na]^+^ expected, 1627.74; found, 1627.93.

**Figure S5** | Smoothed thermal denaturation curves (left) and smoothed first derivative curves (right) for CMPs **1**–**4**. Unsmoothed curves are shown as dashed lines. Parameters used: window_size = 21, poly_order = 1. The concentrations from *A*_205 nm_ measurements were CMP **1**, 47 µM; CMP **2**, 58 µM; CMP **3**, 102 µM; CMP **4**, 92 µM.

| **Table S1** │ *T*_m_ Values^*a*^ (°C) Calculated from the Data in Figure 3B by Different Methods |  |  |  |  |
| --- | --- | --- | --- | --- |
| Method | CMP |  |  |  |
|  | **1** | **2** | **3** | **4** |
| **Smoothed First-Derivative Minimum** | **46.8 ± 0.6** | **40.5 ± 0.0** | **28.8 ± 0.6** | **37.2 ± 0.6** |
| 4-Parameter Sigmoid | 45.3 ± 0.3 | 41.0 ± 0.3 | 21.5 ± 1.1 | 38.2 ± 0.4 |
| 5-Parameter Sigmoid Midpoint | 45.4 ± 0.4 | 41.4 ± 0.2 | 30.9 ± 0.3 | 38.3 ± 0.4 |
| 5-Parameter Sigmoid Inflection Point | 44.9 ± 0.4 | 38.6 ± 0.3 | 26.0 ± 0.3 | 35.8 ± 0.4 |
| ^*a*^Values are the mean ± SD for three replicates. The smoothed first-derivative minimum method selects the temperature among the measured values in the thermal denaturation experiment corresponding to the first-derivative minimum after smoothing, which can coincide across all three samples and result in an SD of 0.0. |  |  |  |  |
